# *EGFR* Exon 20 Insertion Mutations in Sinonasal Squamous Cell Carcinoma

**DOI:** 10.3390/cancers14020394

**Published:** 2022-01-13

**Authors:** Laura Pacini, Virginia N. Cabal, Mario A. Hermsen, Paul H. Huang

**Affiliations:** 1 Division of Molecular Pathology, The Institute of Cancer Research, Sutton SM2 5NG, UK; laura.pacini@icr.ac.uk; 2 Department Head and Neck Cancer, Instituto de Investigación Sanitaria del Principado de Asturias (ISPA), Centro de Investigación Biomédica en Red (CIBER-ONC), 33011 Oviedo, Spain; vncabal@ispasturias.es (V.N.C.); mhermsen@hca.es (M.A.H.)

**Keywords:** EGFR, exon 20 insertions, sinonasal squamous carcinomas, tyrosine kinase inhibitors, drug resistance

## Abstract

**Simple Summary:**

The majority of patients with sinonasal squamous cell carcinoma (SNSCC) associated with inverted sinonasal papilloma carry an exon 20 insertion activating mutation in the epidermal growth factor receptor (EGFR). The aim of this review is to document the various features of EGFR mutations in SNSCC and other cancers, and to assess what we can learn from the study of these mutations in lung cancer, with a special focus on new therapeutic opportunities for SNSCC patients carrying EGFR exon 20 insertions mutations.

**Abstract:**

Recurrent epidermal growth factor receptor (*EGFR*)-activating mutations have been identified in a rare form of head and neck cancer known as sinonasal squamous cell carcinoma (SNSCC), a malignant disease with a 5-year mortality rate of ~40%. Interestingly, the majority of *EGFR* mutations identified in patients with primary SNSCC are exon 20 insertions (Ex20ins), which is in contrast to non-small-cell lung cancer (NSCLC), where the *EGFR* exon 19 deletion and L858R mutations predominate. These studies demonstrate that *EGFR* Ex20ins mutations are not exclusive to lung cancer as previously believed, but are also involved in driving SNSCC pathogenesis. Here we review the landscape of *EGFR* mutations in SNSCC, with a particular focus on SNSCC associated with inverted sinonasal papilloma (ISP), a benign epithelial neoplasm. Taking lessons from NSCLC, we also discuss potential new treatment options for ISP-associated SNSCC harbouring *EGFR* Ex20ins in the context of targeted therapies, drug resistance and precision cancer medicine. Moving forward, further basic and translational work is needed to delineate the biology of *EGFR* Ex20ins in SNSCC in order to develop more effective treatments for patients with this rare disease.

## 1. Introduction

Sinonasal squamous cell carcinoma (SNSCC) is a rare malignancy accounting for 3–5% of head and neck cancer cases and 75% of all sinonasal tumours [1,2,3]. SNSCCs arise from mucosal sites throughout the paranasal sinuses, with the most common originating sites being the nasal cavity and the maxillary sinus [4]. Surgical resection is the treatment of choice in early-stage SNSCC, however, advanced stage tumours can be difficult to resect because of the complexity of the sinonasal anatomy and the proximity to the skull base and orbital cavity [5]. Although surgery with concomitant chemotherapy or radiotherapy have improved the management of this disease, prognosis of patients with SNSCC remain poor, with a 5-year survival rate of approximately 40% [2,3,6,7]. This is in part due to the high rate of local recurrence that has been shown to occur within 2 years of follow-up in 31–56% of cases [5,8,9,10]. Lymph node metastases are relatively infrequent and are found in approximately 10–20% of cases [5,9]. Poor survival is also dependent on the stage of presentation due to often unspecific symptoms such as rhinorrhoea, the difficulty in accessing the tumour due to proximity to vital structures and aetiologic variability.

The aetiology of SNSCC is not fully understood, although several studies have suggested tobacco use, occupational exposures and human papillomavirus (HPV) infection may be risk factors [11,12,13,14]. Another important aetiologic risk for SNSCC is the malignant transformation from inverted sinonasal papilloma (ISP), a locally aggressive benign epithelial neoplasm that arises from the ectodermally derived pseudostratified ciliated (or Schneiderian) epithelium that lines the nasal cavity and sinonasal tract [1,4]. However, SNSCC can also arise as primary malignant tumours without clinical or pathological evidence of an associated papilloma (de novo SNSCC). ISP is the most common sinonasal papilloma subtype based on histological classification (64%), followed by exophytic (ESP, 32%) and oncocytic (OSP, 6%) [15]. These subtypes present fundamental diversities not only based on their histology but also in relationship with their location and viral aetiology. For instance, ESP are tumours driven by low-risk HPV that usually arise in the nasal septum and are only rarely associated with SNSCC. While OSP and ISP share a similar location (the lateral nasal wall and sinuses) and show a similar frequency of malignant transformation in 5–25% and can be associated with synchronous or metachronous SNSCC [12,16,17,18]. Recently, activating mutations in the epidermal growth factor receptor (*EGFR*) and the Kirsten rat sarcoma 2 viral oncogene homolog (*KRAS*) genes have been identified in about 90% of ISP and ISP-associated SNSCC and in 100% of OSP, respectively, but not in ESP or de novo SNSCC [19,20,21]. For the purpose of this review, we will focus on recurrent activating *EGFR* mutations in ISP and ISP-associated SNSCC.

This review was performed by a systematic search of PubMed using keywords relevant to the topic. The most relevant articles and systematic reviews of the two fields discussed (sinonasal squamous cell carcinoma and EGFR exon 20 insertions) were then chosen and reported in the review. The reporting of this review was guided by the standards of the Preferred Reporting Items for Systematic Review and Meta-Analysis (PRISMA) Statement.

## 2. Overview of the Epidermal Growth Factor Receptor (EGFR)

**Figure 1:**
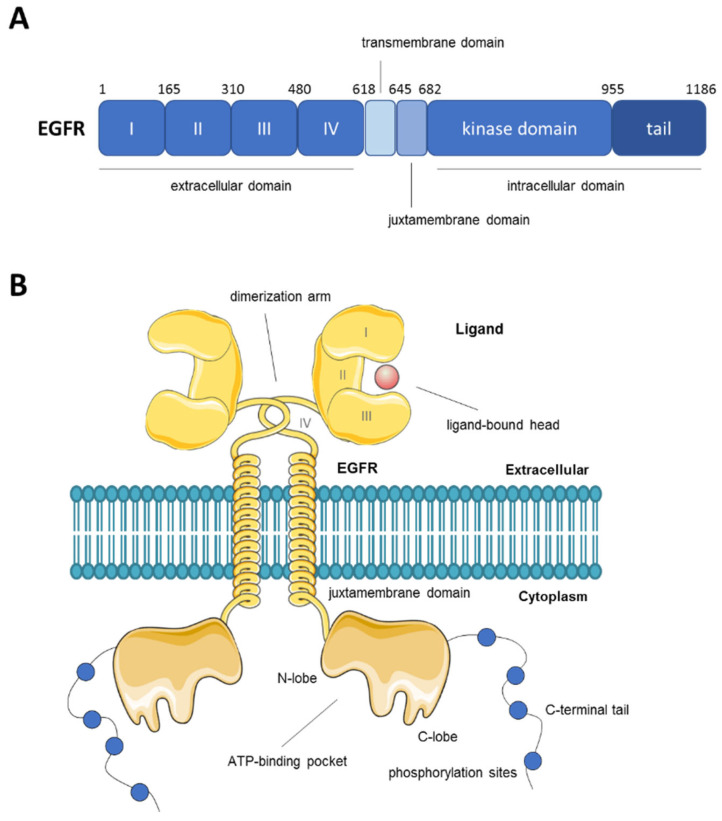
Schematic representation of human EGFR structure and domains. (**A**) Domain boundaries in EGFR with amino acid positions. (**B**) Depiction of the structure of the EGFR dimer in the cell membrane with indication of its various domains and functions.

EGFR is a member of the ErbB family of receptor tyrosine kinases (RTK), which also includes HER2 (ErbB2), HER3 (ErbB3) and HER4 (ErbB4). EGFR is a single-chain transmembrane glycoprotein activated by the binding of various ligands including epidermal growth factor (EGF) and transforming growth factor α (TGFα) [22]. Ligand binding promotes receptor dimerization which drives the autophosphorylation and activation of the kinase domain inducing the phosphorylation of tyrosine residues on the C-terminal tail of EGFR, which act as docking sites for downstream signalling proteins [23]. A schematic representation of EGFR structure and domains is shown in Figure 1. Some of the main signalling pathways triggered by EGFR activation include the rat sarcoma virus (RAS) and mitogen-activated protein kinase (MAPK), phosphatidylinosinol 3-kinase/protein kinase B (PI3K/AKT), phospholipase C-gamma (PLC) and signal transduction and activation of transcription (STAT) pathways. The activation of these pathways regulates cell survival, proliferation and differentiation [24,25]. EGFR plays a crucial role in the physiological regulation of epithelial tissue development and homeostasis [26,27]. In the pathological setting, it is considered an important driver of tumorigenesis, widely studied mostly in lung and breast cancer and glioblastoma (GBM) [28,29].

## 3. EGFR Mutations in Cancer

Given that EGFR activates signalling networks associated with promoting cell survival, growth, invasion and proliferation, it is unsurprising that aberrations that result in hyperactivation of EGFR are common in many cancers. This has led to the extensive investigation of EGFR as a therapeutic target. The mechanisms by which EGFR becomes oncogenic are numerous, mainly resulting from amplification and point mutations at the genomic locus giving rise to constitutively active variants. Ligand overproduction, transcriptional upregulation, defective downregulation of EGFR and cross-talk with heterologous receptor systems have also been described [30]. *EGFR* mutations, amplifications or in-frame deletions can occur in regions corresponding to the extracellular or intracellular portions of the protein and are quite specific to different cancer types.

For instance, in GBM, *EGFR* is among the most commonly altered genes and mutations occur mainly in the extracellular domain [28,30,31]. A large in-frame deletion in the extracellular domain of EGFR spanning exons 2–7 which encode domains I and II, known as *EGFR*vIII, is the most common and well-studied GBM-associated EGFR mutant [32], although less common point mutations in the extracellular domain have also been identified in GBM [31,33,34]. Preclinical studies have shown a certain degree of sensitivity against EGFR tyrosine kinase inhibitor (TKI) therapy, especially the ATP-irreversible second- and third-generation inhibitors, neratinib and osimertinib, respectively [35,36]. Clinical trials are currently ongoing to evaluate the activity of these inhibitors on GBM patients carrying *EGFR*vIII mutations [35,37].

Traditionally, non-small-cell lung cancer (NSCLC) can be classified based on histological differences into adenocarcinoma (~50%), squamous cell carcinoma (~20%) and large cell carcinoma (~3%). The majority of lung adenocarcinomas and squamous cell carcinomas have known oncogenic driver mutations. *EGFR* mutations represent the second most common oncogenic driver event in lung cancer and accounts for ~15–20% of lung adenocarcinoma cases, while *EGFR* mutations are rare in other NSCLC subtypes [38,39]. These mutations are generally restricted to four exons (exons 18–21) in the intracellular kinase domain. The two most common mutations, in-frame deletions in exon 19 (Ex19del) affecting the amino acid motif LREA (E746–750del) and substitution of arginine for leucine at position 858 (L858R) in exon 21, are referred to as “classical” *EGFR* mutations and together account for approximately 85% of *EGFR* mutations in patients with NSCLC [40]. These mutations can result in the constitutive activation of signal transduction pathways, leading to cell proliferation and survival regardless of the presence of extracellular ligands. NSCLC patients with these activating *EGFR* mutations are associated with high response rates to EGFR TKIs, including the competitive, ATP-reversible first-generation inhibitors (gefitinib and erlotinib) and the irreversible second- and third-generation inhibitors (afatinib and osimertinib, respectively) [41].

Exon 20 insertions (Ex20ins) are the third most common *EGFR* mutations to occur in NSCLC after L858R and Ex19del [42,43]. Ex20ins comprise a heterogeneous range of in-frame insertions or duplications that account for 4–10% *EGFR* mutations in NSCLC [42,44,45,46,47]. Similar to Ex19del, there are differences in the exact size and position of the insertion, which ranges from 1–7 amino acids (762–774 amino acid position) most commonly in the loop that follows the αC-helix [46,47]. Recent 3D modelling studies have demonstrated that these mutations lead to a reorganization of critical amino acid residues, which in turn stabilizes ATP binding leading to increased tyrosine kinase activity in the absence of ligand binding [46]. The majority of NSCLC patients harbouring *EGFR* Ex20ins are resistant to clinically approved first- and second-generation EGFR TKIs with low response rates of between 0 and 27% and a median progression-free survival (PFS) of <3 months [48,49,50,51]. Three-dimensional modelling suggests that significant structural alterations caused by the Ex20ins result in a restricted size of the ATP binding pocket, limiting the binding of first-generation EGFR TKIs [52]. In recent years, a new generation of compounds capable of selectively targeting EGFR Ex20ins have been evaluated in a number of clinical trials involving NSCLC patients and two new targeted therapies have recently been approved by the US Food and Drug Administration (FDA) for the treatment of this previously undruggable molecular subtype of NSCLC.

## 4. EGFR Mutations in SNSCC

**Figure 2:**
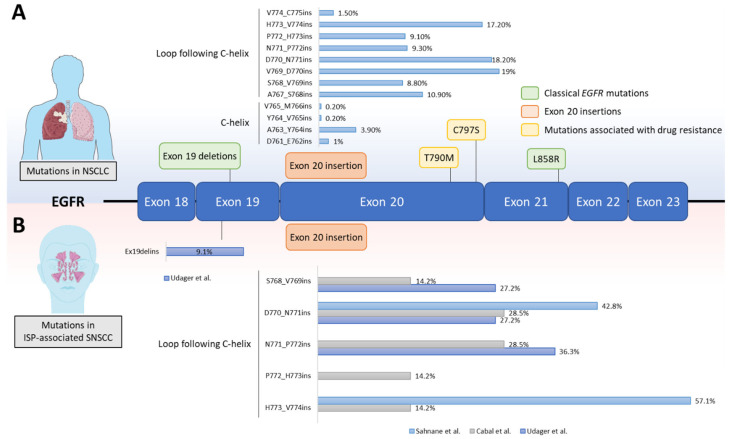
*EGFR* mutations in NSCLC and ISP-associated SNSCC. A schematic representation of the *EGFR* kinase domain is shown. (**A**) All *EGFR* mutations in NSCLC are clustered across exon 18–22, which encode the tyrosine kinase domain. The most common *EGFR* mutations in NSCLC are referred to as ‘classical’ *EGFR* mutations (green) and account for approximately 85% of all *EGFR* mutations in NSCLC patients. The point mutations T790M and C797S (yellow) are associated with resistance to first- and third-generation EGFR TKIs, respectively. The prevalence of exon 20 insertions (orange) in NSCLC that occur at different amino acid positions are shown in the bar chart. Mutation frequency distribution was calculated using COSMIC v86 (http://cancer.sanger.ac.uk, 10 November 2021) after filtering for NSCLC adenocarcinoma patients with exon 20 insertions (*n* = 383) [55]. (**B**) Frequency of exon 20 insertions and exon 19 deletion-insertion in ISP-associated SNSCC across 3 different studies are shown in the bar charts [19,20,56].

While ISPs are considered benign, these tumours have a high rate of local recurrence and can be highly invasive leading to facial deformities and even death in some cases [53]. Moreover, approximately 10–25% cases of ISP are associated with synchronous or metachronous SNSCC [53]. However, the pathogenesis and molecular mechanisms driving oncogenicity in these tumours were unknown until recently. In 2015, a study from Udager et al. investigated the presence of pathogenic somatic mutations by performing next-generation sequencing using a targeted mutation hotspot panel (Ion AmpliSeq Cancer Hotspot Panel) on formalin fixed, paraffin-embedded (FFPE) archival tissues comprising nine ISP, four ISP-associated SNSCC and three non-ISP-associated SNSCC [19]. In this study, the authors identified a high prevalence of *EGFR* mutations in the ISP (7/9) and ISP-associated SNSCC (3/4) cases, but no *EGFR* mutations were observed in the non-ISP-associated SNSCC (0/3) samples. Importantly, this was the first ever study that specifically evaluated *EGFR* mutations—which are rare in head and neck cancer overall—in SNSCC and ISP [54]. In the same study, *EGFR* Exon 18 to 21 was subjected to further analysis by Sanger sequencing in a cohort of FFPE tissue samples from 50 ISP patients and from 22 patients with ISP-associated SNSCC [19]. A total of 19 different *EGFR* mutations were identified in 88% (44 of 50) ISP and 77% (17 of 22) ISP-associated SNSCC tumours. Only one mutation was identified in each tumour and all mutations were confirmed to be somatic by Sanger sequencing of DNA from both tumour and matched normal tissue. Interestingly, the majority of identified *EGFR* mutations were Ex20ins (96% of ISP and 88% of ISP-associated SNSCC), involving residues located between A767 and V774. The most frequent *EGFR* Ex20ins found in the ISP tissues were S768_D770 duplication (S768_D770dup) (25%) and N771_H773dup (18%). Notably, the N771_H773dup was also the most frequent mutation in the ISP-associated SNSCC (29%) followed by S768_D770dupSVD (24%). The other non-Ex20ins mutations found were an *EGFR* deletion-insertion in exon 19 (E746_S752delinsT) in 1/44 ISP case and 2/17 ISP-associated SNSCC and a nucleotide substitution in exon 19 (L747P) in 1/44 ISP. It is interesting to note that the frequency of *EGFR* mutations in these SNSCC tumours is distinct from NSCLC, where Ex20ins are rare, while the Ex19del and L858R mutations predominate. A schematic representation of *EGFR* mutations in NSCLC and ISP-associated SNSCC is shown in Figure 2.

Furthermore, identical *EGFR* genotypes were observed in 12 patients with an ISP and an associated SNSCC when the DNA from each tumour was separately extracted, providing the first molecular evidence to support the role of ISP as a precursor for SNSCC [19]. These results were confirmed in later studies conducted by different groups [20,56,57,58,59,60]. In one study, genotyping analysis for hot-spot mutations in 10 genes (*KRAS*, *EGFR*, *BRAF*, phosphatidynositol-4, 5-bisphosphate 3-kinase catalytic subunit alpha (*PIK3CA*), *NRAS*, anaplastic lymphoma receptor tyrosine kinase (*ALK*), *ERBB2*, discoidin domain receptor tyrosine kinase 2 (*DDR2*), *RET* and *MAPK21*) was performed in a cohort of 65 FFPE specimens from 54 patients distributed as follows: 25 ISP (18 patients), 5 OSP, 24 ISP-associated SNSCC (19 patients) and 12 de novo SNSCC [20]. ISP showed *EGFR* mutations in 18/25 samples (72%), while the remaining samples were wild type for all the genes. Among SNSCC samples, *EGFR* mutations were identified in 2/12 de novo SNSCC (17%) and in 7/23 ISP-associated SNSCC (30%). A total of seven different *EGFR* mutation types were identified, which were mainly Ex20ins involving the D770-V774 region [20]. Again, identical *EGFR* genotypes were observed in paired ISP and ISP-associated SNSCC from 6/7 patients, confirming the existence of a clonal relationship between ISP and synchronous and metachronous SNSCC [20]. Moreover, Cabal et al. have also reported the presence of frequent *EGFR* Ex20ins in 7/18 (38%) ISP and 6/12 (50%) ISP-associated SNSCC but hardly any in de novo SNSCC (5%, 1/19 samples), confirming previous studies [56]. All mutations exclusively concerned in-frame insertions affecting the region S768-V774 in exon 20, with 2 new mutations not previously described in the literature, N771delinsGS in one ISP specimen and P772_H773dupPDN in one de novo SNSCC sample [56]. A list of *EGFR* Ex20ins detected in SNSCC across the different studies is reported in Table 1.

The frequency of *EGFR* mutations in ISP (39%) in the study of Cabal et al. [56] was notably less than in previous studies that reported 72–91% of tumours harbouring mutations [19,20,57,59,60]. Among the ISP-associated SNSCCs, the reported frequency of *EGFR* Ex20ins also differs across different studies: 50% in the Spanish cohort [56], 30% in the Japanese cohort [57] and 72–77% in the Italian and American studies, respectively [20,59]. A possible explanation for these differences could involve the geographic or ethnic factors, which has been observed in the enrichment of *EGFR* mutations in East Asian populations in lung cancer, but not enough evidence is available to support this hypothesis in head and neck cancer [57]. Finally, immunohistochemical expression of EGFR was also reported in a high proportion of ISP (92%, 34/37), ISP-associated SNSCC (60%, 6/10) and de novo SNSCC (44%, 24/54), while the activated phosphorylated form pEGFR was observed in 21/39 (54%) ISP, 4/12 (33%) ISP-associated SNSCC and 20/52 (38%) of de novo SNSCC [56]. Interestingly, the activation of EGFR either through genetic mutations or protein phosphorylation appeared inversely correlated, with 3/12 (25%) *EGFR* mutated cases versus 18/27 (67%) *EGFR* wild-type cases also expressing pEGFR, suggesting that the activation of EGFR pathway may occur through different mechanisms in ISP-associated SNSCC and de novo SNSCC that have reported 50% and 5% Ex20ins mutations, respectively [56]. This may be explained by the fact that Ex20ins mutations lead to a protein conformation change that activates signalling independent of receptor dimerization and autophosphorylation [61].

In conclusion, all these findings in SNSCC open up the possibility for therapy with specific EGFR inhibitors. In the next section, we will review the current literature for previous generation and newly developed compounds that are able to target EGFR Ex20ins and discuss the advantages and disadvantages for the use of these targeted therapies in the treatment of SNSCCs based on the extensive clinical experience gained in the context of lung cancer.

## 5. EGFR Ex20ins Targeted Therapies

Treatment for SNSCC varies depending on the stage, comorbidities and tumour type [5,62,63]. The most common approach is surgical resection with postoperative radiotherapy. Endoscopic surgery is being increasingly used because less invasive, successful management of this disease remains an unmet clinical need, particularly for advanced stage tumours that have infiltrated the hard palate, external maxillary wall, orbital fat or extraocular muscles, orbital apex, brain or facial soft tissue [5,64]. Overall, the prognosis for SNSCC is poor, averaging ~40% at 5 years [65]. Although there is no strong data in the literature, patients with de novo SNSCC have almost a twofold increase in mortality compared with those with ISP-associated SNSCC [17,66,67], but this does not translate into any meaningful difference in treatment management.

The fact that a large proportion of ISP-associated SNSCC patients carry *EGFR* Ex20ins mutations holds promise for new treatment options with EGFR-targeted therapy. In NSCLC, the majority of patients with Ex20ins are resistant to first- and second-generation EGFR TKIs, in contrast to lung cancers with Ex19del or L858R mutations [51,68,69]. Similarly, third-generation inhibitors (such as osimertinib and rociletinib) have shown limited efficacy in pre-clinical and clinical studies in NSCLC patients with *EGFR* Ex20ins who have failed standard platinum chemotherapy [52,70,71]. Despite the general lack of efficacy of first-, second- and third-generation EGFR TKIs, it is notable that small subgroups of Ex20ins patients may benefit from treatment with these agents. For example, it has been shown that NSCLC patients with the rare A763_Y764insFQEA mutation remain sensitive to erlotinib [46]. In the context of SNSCC, Udager et al. have investigated the potential utility of two reversible first-generation inhibitors (gefitinib and erlotinib) and three irreversible second-generation inhibitors (neratinib, afatinib and dacomitinib) in two cell lines derived from SNSCC associated with ISP, SCCNC4 and UM-SCC-112, which carry an S768_D770dupSVD and an N771_H773dupNPH in exon 20, respectively [19]. The authors showed that while ISP-associated SNSCC cell lines were relatively resistant to reversible EGFR inhibitors (IC50 values between 913 nmol/L and >10,000 nmol/L), irreversible inhibitors, and especially neratinib, had a much more potent growth inhibition (as low as IC50 143 nmol/L) [19]. This result is even more striking when the two ISP-associated SNSCC cell lines are compared to a de novo SNSCC cell line, UM-SCC-33 (*EGFR* wild-type), that was fully resistant to all the EGFR inhibitors (IC50 > 10,000 nmol/L) [19]. Moreover, neratinib treatment strongly inhibited EGFR activation in SCCNC4 by reducing the phosphorylation of EGFR and its downstream signalling mediators, such as MAPK and AKT [19]. Interestingly, second-generation inhibitors, and in particular the EGFR/HER2 irreversible inhibitor dacomitinib, were also shown to achieve a variable degree of growth inhibition in a panel of *EGFR* Ex20ins engineered Ba/F3 and NIH-3T3 cell lines [72]. In this study, the authors identified the insertion of glycine at position 770 as a common feature among the dacomitinib-sensitive mutations. Moreover, data from a phase I clinical trial (NCT00225121) showed a partial response to dacomitinib in one NSCLC patient with a D770delinsGY in exon 20 supporting the hypothesis of a relationship between specific mutations and corresponding drug sensitivity [73]. Further studies are needed to corroborate these data and provide biological insights into the relationship between drug efficacy and the molecular heterogeneity of Ex20ins mutations in SNSCC patients.

The mutations that have shown a response to first- and second-generation EGFR TKIs previously described are generally rare in SNSCC (as well as in NSCLC), and therefore the majority of EGFR Ex20ins patients are unlikely to benefit from these therapies. In recent years, next-generation agents that are able to more selectively target Ex20ins mutants have undergone pre-clinical studies and clinical trials to evaluate their safety and efficacy in NSCLC patients carrying these mutations. Here, we outline the most clinically advanced candidates that could provide valuable therapeutic options for SNSCC patients harbouring *EGFR* Ex20ins mutations.

In September 2021, the FDA granted accelerated approval to mobocertinib (TAK-788) for patients with locally advanced or metastatic NSCLC with *EGFR* Ex20ins mutations whose disease has progressed on or after platinum-based chemotherapy. Mobocertinib is a covalent, irreversible inhibitor that specifically targets EGFR and HER2 [74]. Unlike previously approved reversible and irreversible inhibitors, mobocertinib has shown the ability to bind and inactivate the compact ATP-binding site of EGFR Ex20ins mutants that was shown to be inaccessible to most compounds [75]. Approval was based on results from an international, non-randomized, open-label, multicohort EXCLAIM clinical trial (NCT02716116), which evaluated mobocertinib efficacy in a cohort of 114 NSCLC patients carrying *EGFR* Ex20ins mutations that have progressed on or after platinum-based chemotherapy [76,77,78]. Patients were administered with mobocertinib 160 mg orally daily until disease progression or intolerable toxicity. The overall response rate (ORR) was 28% with a median PFS of 17.5 months. The most common side effect (>20%) included diarrhoea, rash and nausea.

Amivantamab, an EGFR and hepatocyte growth factor receptor (MET)-targeted bispecific antibody, has also shown promising efficacy in a multicentre, non-randomized, open label, multicohort CHRYSALIS clinical trial (NCT02609776) involving 81 patients with advanced NSCLC carrying *EGFR* Ex20ins mutations that have progressed after previous treatment [79]. Patients were treated with amivantamab 1400–1050 mg (based on body weight) once weekly for 4 weeks, then every 2 weeks until disease progression or unacceptable toxicity. The ORR was 40% with 11.1 months median PFS. The safety profile was manageable. Based on these results, in May 2021, the FDA granted accelerated approval for amivantamab in NSCLC patients with *EGFR* Ex20ins whose disease has progressed on or after platinum-based chemotherapy.

Poziotinib (HM781–36B), an irreversible EGFR inhibitor, has also been assessed in a number of phase II clinical trials (NCT03066206, NCT03318939) in patients with NSCLC with Ex20ins mutations. As for mobocertinib, poziotinib has shown the ability to access the restricted drug-binding pocket of Ex20ins mutants [52]. However, unlike classical *EGFR* mutations, Ex20ins mutations can activate EGFR without diminishing ATP affinity versus the wild-type kinase [46]. Indeed, poziotinib is also a potent inhibitor of wild-type EGFR [52], raising concerns that poziotinib may show a narrow therapeutic window, linked to insufficient therapeutic dosing due to toxicity and therefore contributing to short-term tumour responses. In March 2021, the FDA granted poziotinib breakthrough designation status based on promising responses seen across these trials. At that time, there were no approved therapies for the treatment of Ex20ins NSCLC patients. Despite encouraging pre-clinical and clinical results, toxicity remains a major concern for this drug. For instance, in the phase II, open-label, multi-cohort, multicentre ZENITH20 trial (NSCT03318939), a cohort of 115 NSCLC patients that have progressed after previous treatment with a proven *EGFR* or *HER2* Ex20ins mutation who were treated with 16 mg poziotinib once daily achieved an ORR of 14.8% and a disease control rate (DCR) of 68.7% with a median PFS of 4.2 months [80]. Grade 3–4 adverse events were reported in 63% of patients, most commonly diarrhoea and skin rash. As a result, 68% of patients required dose reductions to subtherapeutic doses. These data highlight the limited clinical efficacy of poziotinib and the challenge of targeting *EGFR* Ex20ins without significant toxicity due to concurrent wild-type inhibition.

Other therapeutics with the ability to target Ex20ins are the covalent, irreversible inhibitor TAS6417 (CLN-081) [81] and the heat shock protein 90 (Hsp90) inhibitor luminespib (NVP-AUY922) [82]. TAS6417 has shown selectivity for Ex20ins over *EGFR* wild-type in preclinical cell line models indicating a promising wider therapeutic window to target Ex20ins mutants [81]. Luminespib has also shown anti-tumour activity in preclinical studies [83]. Preclinical studies and clinical trials are currently evaluating these compounds in NSCLC patients harbouring *EGFR* Ex20ins mutations [82,84]. Preliminary results from a phase II clinical trial (NCT04036682) evaluating TAS6417 in a cohort of 37 NSCLC patients with *EGFR* Ex20ins previously treated with platinum-based therapy showed an acceptable safety profile and encouraging anti-tumour activity [84]. The most common adverse events were rash and diarrhoea, with four patients requiring dose reduction and two patients having to discontinue treatment due to hypersensitivity reactions and pneumonitis [84]. Among the 25 patients with evaluable response, 10 (40%) had a partial response, 14 (56%) had stable disease and 1 (4%) had progressive disease. Of the patients with partial response and stable disease, 20/24 (83%) had tumour regression [84]. Luminespib has been evaluated in a phase II clinical trial (NCT01854034) that enrolled patients with late-stage NSCLC carrying *EGFR* Ex20ins that have progressed after previous treatment [82]. The ORR among the 29 patients was 17% with a median PFS of 2.9 month [82]. Although luminespib is generally well-tolerated, reversible low-grade ocular toxicity is common. Taken together, the clinical data to date highlight the challenges of targeting Ex20ins mutations with toxicity due to wild-type EGFR inhibition and the high heterogeneity of this class of mutations remaining the main limiting factors.

The potential for combination treatment is a key area of interest. Combinations of antibody-TKI currently being investigated for the treatment of *EGFR* Ex20ins NSCLC patients include cetuximab, necitumumab and amivantamab in combination with different second- and third-generation EGFR TKIs. Cetuximab is a monoclonal antibody (mAb) that binds to EGFR extracellular domain, preventing ligand binding and blocking receptor activation [85]. After promising results in preclinical models [86], an afatinib and cetuximab combination achieved a partial response (2.7 to 17.6 months) in 3/4 NSCLC patients with *EGFR* Ex20ins previously treated with platinum-based chemotherapy [87]. A phase II single-arm clinical trial evaluating afatinib and cetuximab in *EGFR* Ex20ins advanced NSCLC patients is currently ongoing (NCT03727724). Preliminary results from 18 enrolled patients in this trial demonstrated substantial antitumour activity of the afatinib and cetuximab combination with a disease control rate of 59% at 18 weeks and a response rate of 47%, with manageable toxicity [88]. Similarly, a recent phase I dose escalation study (NCT02496663) evaluating necitumumab, a mAb that binds the extracellular domain of EGFR preventing receptor-ligand binding activation and inducing receptor internalisation [89], and osimertinib in patients with advanced NSCLC and resistant to prior EGFR TKI therapy has shown responses in 2/4 patients carrying Ex20ins, with a median PFS of 5.3 months [90]. In addition, amivantamab is also currently being evaluated in combination with the third-generation EGFR inhibitor lazertinib as part of the combination and dose-expansion cohort of the CHRYSALIS phase I trial (NCT02609776), but no results have been reported yet [91]. The results of these trials are eagerly awaited to determine the impact of combination mAb and TKI therapy on *EGFR* Ex20ins tumours.

## 6. Lessons from Lung Cancer That Could Be Applied to the Treatment of SNSCC

The use of EGFR TKIs has improved the outcomes of NSCLC patients carrying *EGFR* Ex20ins mutations. This has generated interest in the possibility of a similar impact of EGFR targeted therapy in *EGFR*-mutant SNSCC. Several outstanding questions, however, remain to be addressed. For instance, as described above, many of the compounds that target *EGFR* Ex20ins mutants have limited clinical efficacy, highlighting the challenges associated with the significant molecular heterogeneity of this class of mutations (Figure 2). A recent study from Robichaux et al. has reported an alternative way of predicting patient outcomes following treatment with EGFR inhibitors [68]. This is based on structural and functional changes that are induced by specific mutations [68]. This approach was shown to be more powerful at predicting drug sensitivity compared to the traditional exon-based classification. The authors analysed a panel of 76 cell lines harbouring *EGFR* mutations (spanning exons 18 to 21), which was subjected to treatment with 18 different EGFR TKIs (including first-, second-, third-generation and Ex20ins TKIs). They found that Ex20ins mutants could be divided into two subgroups based on differential drug sensitivity: (i) classical-like mutations that were distant from the ATP-binding pocket (insertions in the αC-helix) and (ii) insertions in the loop at the C-terminal end of the αC-helix (Ex20ins-L) [68]. Insertions in the αC-helix showed high sensitivity to all the inhibitors tested, while Ex20ins-L mutations were sensitive only to selective TKIs (poziotinib and TAS6417). Moreover, even within the Ex20ins-L mutations, some degree of heterogeneity was observed that allowed a further sub-classification into near- and far-loop Ex20ins mutants, with the near-loop mutations showing more sensitivity to second-generation and Ex20ins TKIs compared to the Ex20ins far-loop mutants [68]. Given these data, this structure-based approach to classification of drug response has the potential to improve the prediction of effective treatment options for patients carrying rare *EGFR* mutations.

With multiple new TKIs and mAb that target *EGFR* Ex20ins under investigation, which drug to give and in which line of treatment remain key questions in clinical management. Currently, the two agents (amivantamab and mobocertinib) used in the clinic for the treatment of NSCLC carrying *EGFR* Ex20ins have been granted approval from the FDA as second-line therapy in patients that have progressed on platinum-based chemotherapy. The trials to evaluate the use of these agents as first-line therapies for the treatment of *EGFR* Ex20ins mutant NSCLC are ongoing. A randomized first-line study of amivantamab plus chemotherapy versus chemotherapy alone in *EGFR* Ex20ins is ongoing (PAPILLON, NCT04538664). Moreover, the phase III EXCLAIM-2 clinical trial is evaluating the efficacy of mobocertinib versus platinum-doublet chemotherapy among treatment-naïve patients (NCT04129502). The outcome of these studies will be particularly useful to inform future clinical studies in the context of previously untreated SNSCC patients who harbour *EGFR* Ex20ins mutations. Another unresolved question is whether the treatment of SNSCC *EGFR* Ex20ins tumours with targeted therapies will achieve durable clinical response or instead will result in the development of acquired resistance mechanisms, as observed in NSCLC patients. Taking lessons from EGFR inhibitor therapy in NSCLC, several mechanisms of resistance can be predicted in the context of SNSCC. The most common mechanisms include on-target *EGFR* secondary mutations, compensatory bypass pathway activation, the acquisition of an epithelial to mesenchymal transition (EMT) phenotype and the presence of drug-tolerant persister (DTP) cells in the heterogeneous tumour population [92]. Specifically, clinical mechanisms of resistance have been reported for some of the Ex20ins inhibitors mentioned before [93]. Evidence from the use of poziotinib in NSCLC patients and in preclinical models suggests drug resistance can be driven by the acquisition of secondary on-target mutations in *EGFR*, such as the T790M gatekeeper and C797S point mutation [52,93], which have been extensively studied in the context of acquired resistance to first- and third-generation EGFR inhibitors, respectively [94,95,96]. Both poziotinib and mobocertinib have shown the ability to covalently bind to the *EGFR* C797 cysteine residue, indicating that point mutations in this amino acid may confer Ex20ins TKIs resistance [52,97]. In addition, co-occurring mutations in *KRAS* and *ErbB4* have also been shown to drive resistance to poziotinib in genetically engineered mouse models harbouring tumours expressing *EGFR* Ex20ins (D770insNPG) [93]. Moreover, mutations in *MAPK2* and *PIK3CA* and amplifications in *MET* and cyclin-dependent kinase 6 (*CDK6*) have also been identified in Ex20ins NSCLC patient biopsies that progressed after poziotinib treatment [93]. Interestingly, *MET* and *CDK6* amplifications are known mechanisms of resistance to first- and third-generation EGFR TKIs and can be targeted to overcome resistance to Ex20ins inhibitors [98,99].

In the context of ISP-associated SNSCC, little is known about the oncogenic mechanisms driving the malignant progression from sinonasal papillomas to carcinoma. A comprehensive assessment of the molecular landscape of mutations and copy number alterations in papilloma-associated SNSCC utilising targeted next-generation DNA sequencing of frequently altered cancer genes was performed by Brown et al. [100]. The authors confirmed the presence of recurrent *EGFR* mutations in 21/24 ISP-associated SNSCC [100]. Interestingly, *EGFR* mutations were mutually exclusive with mutations in *KRAS*, which is commonly found in OSP-associated SNSCC [100]. In addition, recurrent mutations were found in the tumour suppressor *TP53* (in 16/24 cases) and in the cyclin-dependent kinase inhibitor 2A (*CDKN2A)* (in 10/24 cases) in ISP-associated SNSCC [100]. Notably, these inactivating mutations were not observed in the matched ISP tumours suggesting that these alterations may be early molecular events in the malignant progression to carcinoma. Activating mutations (4/24) and copy number gain (2/24) in *PIK3CA* were also shown to co-occur with *EGFR* mutations in ISP-associated SNSCC [100]. These data were confirmed by an independent study from Uchi et al. in a smaller cohort that also reported high prevalence of co-occurring mutations in neurofibromin 1 (*NF1*) in *EGFR* mutated ISP-associated SNSCC tumours [101]. Interestingly, *TP53* and *CDKN2A* have also been identified as common co-occurring alterations in *EGFR* ex20ins NSCLC tumours [102], while a E545K mutation in *PIK3CA* was detected in 1/20 Ex20ins NSCLC patient that progressed after poziotinib treatment [93]. It is plausible that these co-occurring genomic aberrations may play a role in driving intrinsic and acquired resistance to EGFR TKI therapy in SNSCC.

EMT has also been shown to confer resistance to clinically approved EGFR inhibitors that target classical *EGFR* mutations in NSCLC [103,104] and has been indirectly identified as a potential mechanism of resistance to poziotinib in NSCLC cell lines, HCC4006 and HCC827, harbouring classical *EGFR* mutations (Ex19del) [52]. Subjecting these two cell lines to chronic escalating dose treatment led to the development of erlotinib resistance and EMT. These EMT-driven, erlotinib-resistant cells also showed resistance to poziotinib, indicating that poziotinib may be susceptible to similar mechanisms of acquired resistance as other EGFR TKIs that target classical EGFR mutations [52]. Finally, it is now well established that the subpopulation of DTP cells that remain viable in the presence of anti-cancer treatments despite not harbouring classic genetic mutations can contribute to acquired resistance to first- and third-generation EGFR TKIs in NSCLC cell line models [92,105,106,107]. This knowledge from classical *EGFR* mutations in lung cancer is important as it allows us to anticipate the potential routes of drug resistance to EGFR Ex20ins inhibitors in other clinical settings, such as in SNSCC. A better understanding of the resistance mechanisms arising following EGFR TKIs treatment may facilitate the identification of new therapeutics that could be used as salvage therapy.

## 7. Conclusions

The incidence of *EGFR* Ex20ins mutations in ISP and ISP-associated SNSCC tumours implicates a prominent role for activating *EGFR* mutations in these diseases and opens up an exciting opportunity for treatment with EGFR targeted therapies. Although this class of mutations is associated with poorer response to first-, second- and third-generation EGFR TKIs compared to classical *EGFR* mutations, a number of next-generation EGFR targeted agents have recently been approved for the treatment of NSCLC tumours harbouring Ex20ins or are currently undergoing clinical evaluation [79,80,82,84]. However, there is evidence in lung cancer of a poor therapeutic index in some of these inhibitors and rapid development of acquired resistance. Further research is therefore needed to better understand whether SNSCC patients with Ex20ins will respond to these inhibitors but also to develop more insights into the spectrum of biological mechanisms of drug resistance.

## Figures and Tables

**Table 1:** Prevalence and distribution of *EGFR* exon 20 insertions in ISP, ISP-associated SNSCC and de novo SNSCC.

Mutation	Frequency per Tumour Types			Ref.
	ISP	ISP-Associated SNSCC	De Novo SNSCC	
A767_V769dup	2%			[19]
S768_D770dup	25%	24%		[19]
V769_D770insGSV	2%			[19]
D770_P772dup	5%			[19]
D770_N771insGF	2–17%			[19,58]
D770_N771insSVD	5–24%	1–25%		[20,56,58,60]
D770_N771insGD	2%	6%		[19]
D770_N771insSVE	2%	6%		[19]
D770_N771insG	2–6%	2–7%	1%	[19,20,56,58,60]
D770_N771insGL	2–9%	6–7%		[19,56,60]
]N771delinsGS	2%			[56]
N771delinsGF	9%		1%	[19,60]
N771delinsGY	2%	14%		[19,56]
]N771delinsSG	2%			[19]
N771_P772insV	2%	6%		[19,56]
N771_H773dup	18%	29%		[19]
N771_P772insPDN		15%	1%	[60]
P772_H773dupPDN			1%	[56]
P772_H773insDNP	9%			[58]
H773_V774insGCRH	2%			[19]
H773dup	11%			[19]
H773_V774dup	2%			[19]
H773_V774insPH	1%			[20]
H773_V774insH	3–4%	1%		[20,58]
H773_V774insNPH	3–9%	2–45%	1–2%	[20,56,58,60]
